# Osteopontin is a marker for cancer aggressiveness and patient survival

**DOI:** 10.1038/sj.bjc.6605834

**Published:** 2010-09-07

**Authors:** G F Weber, G S Lett, N C Haubein

**Affiliations:** 1 University of Cincinnati Academic Health Center, James L. Winkle College of Pharmacy, 3225 Eden Avenue, Cincinnati, OH 45267-0004, USA; 2 MetaMol Theranostics, Cincinnati, OH, USA; 3 The BioAnalytics Group, Jamesburg, NJ, USA

**Keywords:** metastasis, survival, grade, stage

## Abstract

**Background:**

Only a fraction of molecular cancer markers identified in the scientific literature have found clinical use. Specifically, few predictors of invasiveness are established in diagnostics. Meta-analysis is a valuable tool for biomarker validation. Here, we evaluate Osteopontin as a marker for tumor aggressiveness (grade, stage, early progression) and patient survival.

**Methods:**

Publications through 2008 with the keywords ‘osteopontin AND cancer’ were retrieved. Titles and abstracts were screened for studies presenting original data on human subjects. This left 228 publications for data extraction. We applied categorical data analysis for testing the relationship between Osteopontin and a clinical variable.

**Results:**

Osteopontin ranks correlated with lower overall and disease-free/relapse-free survival in all tumors combined, as well as in lung cancer, breast cancer, prostate cancer, head and neck cancer, and liver cancer. Further, Osteopontin levels correlated with tumor grade and stage for all tumors combined and for several individual tumor types. Osteopontin levels were significantly associated with the early progression of eight cancers, independent in one, and inversely correlated in two.

**Conclusions:**

Osteopontin is significantly associated with survival in several forms of cancer. Osteopontin levels are also markers for stage, grade, and early tumor progression in multiple cancers, reflecting a common molecular underpinning for distinct clinical measures. Osteopontin has value as a clinical tumor progression marker.

In recent years, substantial progress has been made in the detection and diagnosis of early stage cancers. This is mostly due to improved imaging technologies and new biomarkers in histological and hematologic testing. However, there still is a dearth of molecular indicators that distinguish highly aggressive tumors from moderately aggressive and non-aggressive ones. Specifically, few markers that predict invasiveness have been firmly established. Better molecular prognostics are needed to accurately assess disease. One candidate marker for the progression of various malignant tumors has been Osteopontin. In cancer, this molecule can support cell invasion and anchorage independence, thus enhancing tumor progression and metastasis formation (Weber, 2008). Despite a large literature on Osteopontin as a cancer marker, it is not in routine diagnostic use. One reason may be the diversity of source materials and cancer-associated readouts that have been investigated in correlation to Osteopontin levels. Therefore, it is important to analyze the comprehensive published evidence to discern which aspects of cancer pathophysiology are consistently associated with elevated Osteopontin levels, thus validating this molecule as a candidate marker.

The scientific literature on biomarkers has grown disproportionately more rapidly than the application of promising markers in clinical practice. Among the reasons for the delay are high barriers in the regulatory process and limited available resources for the recruitment and analysis of sufficiently large patient populations. Meta-analysis is a suitable approach to enhancing knowledge about the diagnostic potential of individual biomarkers within these confines. Yet, conventional regression algorithms have had limited capability of combining distinct data sets and have therefore often fallen short of improving confidence. This is a particular problem for immunohistochemistry, where variable staining protocols combined with the semi-quantitative nature of the examination generate substantial study-to-study fluctuations. Categorical data analysis can limit such heterogeneity. The evaluation of within-study ranks results in a self-normalization of variable data sets. When applied to the meta-analysis of biomarkers, categorical data analysis has a dramatically higher sensitivity than conventional regression algorithms for detecting trends in data sets from disparate sources.

## Methods

### Data extraction

A PubMed search with the keywords ‘osteopontin AND cancer’ through December 2008 resulted in 800 hits. Titles and abstracts were screened for studies involving human subjects, yielding 271 papers for initial analysis. 36 articles (including reviews, commentaries, experiments only on cell lines, no results on cancer, etc.) did not contain new data on Osteopontin in human cancer. Four articles were not obtained, even after request through interlibrary loan. Three papers were excluded because they contained one retraction, one article that pooled diverse primary tumors without separating them by tumor type, and one paper that applied scientifically questionable methodology (bidigital O-ring test). This left 228 publications to be used for data extraction (Table 1). Of articles not written in English, only the abstracts (not the full texts) were drawn on for obtaining data. For data extraction, numbers from the article text were applied directly; data presented in the format of graphs were measured and converted to the relevant units. Data from Kaplan-Meier survival curves were digitized using the software DataThief.

The cancers covered by the original publications include: breast cancer (34), ovarian cancer (25), liver cancer (21), lung cancer (20), head and neck cancer (15), colorectal cancer (14), gastric cancer (14), prostate cancer (13), bone cancer (9), oral cancer (9), melanoma (9), pancreatic cancer (8), renal cancer (8), esophageal cancer (7), glioma (7), mesothelioma (7), thyroid cancer (7), endometrial cancer (6), myeloma (6), cervical cancer (4), gestational trophoblastic tumor (4), leukemia/lymphoma (3), granular cell tumor (2), non-melanoma skin cancer (2), ampullary cancer (2), bladder cancer (2), medulloblastoma (2), soft tissue tumors (2), teratoid tumor (2), adrenocortical cancer (1), neuroblastoma (1), pilomatricoma (1), renal pelvis cancer (1), von Hippel-Lindau disease (1). The numbers in parentheses indicate the number of publications for each type of cancer. Note that several papers contain data on more than one type of cancer and are counted here for each. Therefore the sum is larger than the 228 original publications used for the data extraction.

### Data analysis

A significance level of 95% (*P*<0.05) was applied to all studies. The correlation between Osteopontin expression levels and the clinical variables of interest was examined with a categorical approach (using ranked values). Within a study, the clinical variables were ranked from low to high and then normalized by the number of examples in the study. Studies that combined a range of grades were assigned the mean grade. Also within a study, the Osteopontin scores were ranked from low to high. In the case of immunohistochemistry scores that reported graded results on a 0–3^+^ scale, a composite score for the study was computed by weighting each score by the fraction of patients reported for that score. For studies using an expanded scoring system, the scores were grouped at low, medium, and high levels and treated in the same way as the 0–3^+^ results. For studies that only reported mean or median results, the raw values were simply ranked. Ranking accomplishes a self-normalization within each study (Hong *et al*, 2006; Hong and Breitling, 2008) and permits the simultaneous analysis of both the summary results (mean, median only) and various graded results. In the case of immunohistochemistry, this reduces the effects of different pathologists scoring the samples. In other assay types, such as ELISA or quantitative RT-PCR, this eliminates the need for a normal standard under the assumption that all samples within a study are compared against the same standard.

We utilized the Pearson *χ*^2^ test (Agresti, 2007) for independence to assess whether the Osteopontin ranks are independent of the clinical variable ranks. This test was carried out by constructing contingency tables using the ranks for each variable, and populating each cell with the total number of patients reporting that combination of ranks. Separate tables were constructed for sets of studies with 2, 3, or more ranks to avoid structural zeros. The Mantel-Haenszel *χ*^2^ test (Agresti, 2007) was used to assess the hypothesis that the ranking of a particular clinical variable within a study is linearly related to the Osteopontin level. We then tested for a non-linear trend by examining the residuals between the observed values and a linear model of the data.

Receiver operator characteristic (ROC) curves are commonly used to assess diagnostic performance of a particular measurable quantity. The most common feature used to quantify this characteristic is the area under the curve, which can be interpreted here as the probability that for two randomly chosen samples, the one with the higher Osteopontin rank will also have a higher rank for the clinical variable in question (Rice and Harris, 2005). In the case of the ranked data in this study, that probability can be calculated for each clinical study. Each pair of patient groups in the study was examined, and the fraction of those where a group with higher clinical variable rank also had a higher Osteopontin level rank is reported here. The statistical significance of this fraction was tested by carrying out a Monte Carlo simulation to estimate the distribution of fractions expected for random ranks.

### Reporting standards

The data applied to this study were not skewed by publication bias according to a funnel plot analysis. The present study has been conducted according to the standards of the PRISMA Statement (Moher *et al*, 2009).

## Results

### Osteopontin in patient survival

**Figure 1:**
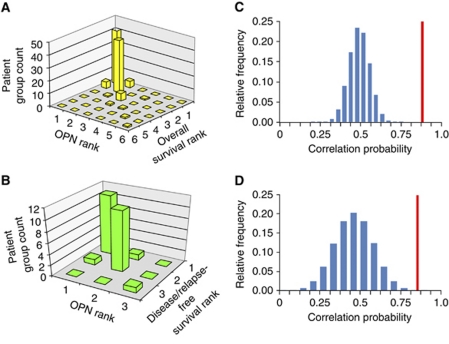
Osteopontin in overall survival and in disease-free/relapse-free survival. (**A**) Overall survival and Osteopontin ranks for all cancers combined. (**B**) Disease-free survival and Osteopontin ranks for all cancers combined. (**C**) Probability Distribution Function for independent Osteopontin and overall survival ranks. The measured value for Osteopontin data is shown as a vertical line. (**D**) Probability Density Function for independent Osteopontin and disease free survival ranks. Measured value for Osteopontin data is shown as a vertical line.

We applied categorical meta-analysis to the evaluation of Osteopontin as a prognostic marker. The distribution of ranks for published overall and disease-free/relapse-free survival versus measured Osteopontin levels displayed an aggregation along the diagonal in bar graphs, indicating a good correspondence for higher Osteopontin rank to lower mean survival time (Figure 1A and B). To further quantify these results, we determined the probability that for two patient groups selected at random from a study, the one that had the higher Osteopontin score would also have a shorter mean survival time. This resulted in a probability of 90.8%, *P*<1 × 10^−5^ for overall survival and a probability of 92.9%, *P*=1 × 10^−4^ for disease-free/relapse-free survival, where the significance was estimated using a permutation test. These results indicate that Osteopontin rank is a good predictor of survival outcome rank within a study. The actual probability calculated from the meta-analysis of the data was significant when compared to the estimated probability distribution under the null hypothesis that Osteopontin and mean survival time are independent (Figure 1C and D). When broken down to individual cancers, the association between Osteopontin levels and overall survival was significant for lung cancer, breast cancer, prostate cancer, head and neck cancer, and liver cancer (Table 2). Similar results were obtained using the meta-analysis function in Oncomine (Supplement 1). For several cancer types, only one published study was available. Those cases were excluded from the meta-analysis.

In clinical practice, the detection of Osteopontin is particularly important in two settings. In serum or plasma, Osteopontin may serve as a prognostic marker associated with a minimally invasive procedure. After a biopsy, Osteopontin may serve as a prognostic marker directly linked to the tumor. Therefore, we separately analyzed the patient survival data for Osteopontin in these distinct types of specimens. For all cancers combined, the levels of Osteopontin in plasma, in serum, and in tumors significantly identified subpopulations with shorter mean survival (Table 3). In tumors, the highest Osteopontin groups had a mean survival 850 days shorter than the lowest Osteoponin groups. For plasma, the highest Osteopontin groups had a mean survival 560 days shorter than the lowest Osteoponin groups. The concordance between Osteopontin ranks and risk for reduced survival was confirmed for several individual cancers (Table 3). However, the sample sizes for several individual cancers were not sufficiently large to obtain 95% significance for the rank statistic used here (in plasma for gastric, cervical, liver, teratoid, esophageal, and renal cancers; in serum for breast cancer, head and neck cancer, and mesothelioma; in tumors for colorectal, ovarian, and prostate cancers, mesothelioma, and glioma). In tumors, discordance (i.e. higher Osteopontin groups had longer mean survival times) was observed for one study each on bone cancer, endometrial cancer, and melanoma.

### Osteopontin in tumor grade, stage and early progression

**Figure 2:**
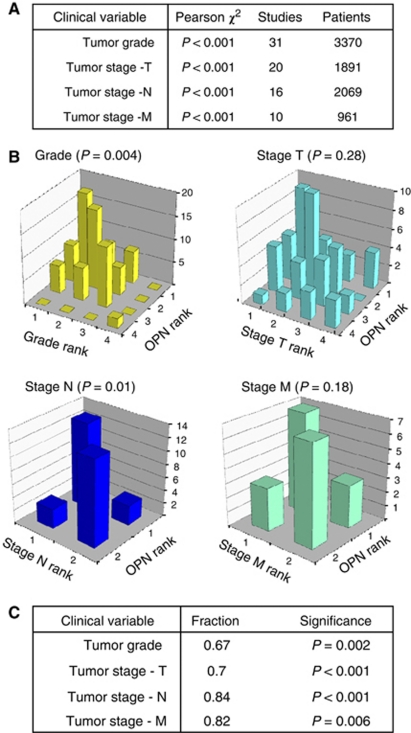
By categorical meta-analysis, Osteopontin levels correlate with stage and grade of cancers. (**A**) The Pearson *χ*^2^ test of ranked Osteopontin immunohistochemistry scores with tumor grade and stage shows a significant dependence between Osteopontin rank and clinical variable. (**B**) The bar graphs of Osteopontin rank versus rank of grade or stage display an aggregation of data along the diagonal, indicating a positive relationship between Osteopontin levels and clinical variables. The associations are statistically significant for grade and node positivity, but not for stage T and M. (**C**) Expanded analysis of grade and stage ranks to all published measures. In five studies with duplicate data sets only the immunohistochemistry results were used. We computed a measure analogous to that represented by the area under a ROC curve (see Methods). For all grade and stage measures, Osteopontin is a significant positive indicator.

Osteopontin immunohistochemistry score ranks and tumor grade and stage ranks were dependent (*P*<0.001) for all cancers combined (Figure 2A), as well as for 12 individual cancers for grade, 13 individual cancers for stage T, 8 individual cancers for stage N, and 9 individual cancers for stage M (Table 4). Graphical representation of the group ranks suggested a strong positive relationship, reflected in a high density of data points along the diagonal in bar graphs (Figure 2B). To further quantify these ranked data, we determined the probability that for two patient groups, the one that had the higher Osteopontin rank would also have a higher grade or stage rank. In 66.3% of these comparisons, the group with higher Osteopontin rank was also the group with a higher tumor grade, which Monte Carlo analysis revealed to be significant (*P*=0.004). Positive comparisons were also seen in 81.3% of cases for tumor stage N (node involvement, *P*=0.01), 54.5% of cases for tumor stage T (primary tumor, *P*=0.28), and 70% of cases with higher tumor stage M (metastasis, *P*=0.18).

For stage T and M, the positive relationship identified in the comparison of ranks was not statistically significant, possibly due to insufficient sample size. Advantageously, the categorical analysis can be applied to heterogeneous data sets. By combining immunohistochemistry with the other published tests, we identified a statistically significant relationship between Osteopontin levels and all grade and stage measures, including T and M, thus demonstrating the benefit of incorporating all of the available data within one analysis (Figure 2C). The categorical analysis had higher sensitivity than a conventional meta-analysis approach (Supplement 2).

In the early stages of transformation, tumor progression can be described as the transition from normal tissue to precancerous lesions (dysplasia, metaplasia), preinvasive cancer, and cancer. According to categorical meta-analysis, Osteopontin expression levels were significantly associated with the progression of eight cancers, independent in one, and inversely correlated in two (skin cancer and gestational trophoblastic tumor) (Table 5). Of note, while Osteopontin appears to be a cancer biomarker for 31 individual malignancies its levels were significantly reduced below normal in non-melanoma skin cancer and gestational trophoblastic tumor. This suggests a unique role for Osteopontin in these two malignancies.

## Discussion

High levels of Osteopontin in several cancers are indicative of a poor prognosis. Overall and disease-free survival are inversely related to Osteopontin levels in several cancers. There is strong correspondence between high Osteopontin and lower mean survival time in tumor (82%) and plasma (100%) measurements, with large mean differences in survival times, indicating a useful role for Osteopontin in patient stratification, Patient survival is largely determined by tumor aggressiveness. Hence, it is not unexpected that Osteopontin, a prognostic measure for survival, is also a marker for grade, stage, and early progression. It is likely that patients with elevated Osteopontin at the time of diagnosis warrant more forceful treatment regimens than are suitable for patients with low levels of Osteopontin.

Although tumor grade, tumor stage, and early tumor progression are distinct measures for the clinical presentation of a cancer they are not mutually unrelated. A dedifferentiated, high grade tumor is more aggressive, and consequently more likely to disseminate and become high stage than a low grade tumor. The molecular mechanisms driving progression, grade, and stage are overlapping. Osteopontin is associated with all of them. In patient care, the diagnosis and assessment of cancer is typically made on the basis of clinical and histo-morphologic criteria. However, molecular markers are more quantifiable and may be more reflective of underlying disease mechanisms. The incomplete convergence between clinical and molecular descriptors may require a reevaluation of how we assess cancer (Weber, 2010).

In this analysis, the concordance between Osteopontin expression rank and stage or grade rank was 67–84% over all types of cancer. This is comparable to the accuracy commonly estimated for existing tumor markers, including CEA, CA 15-3, CA 19-9 and PSA (Ebert *et al*, 1996; Koopmann *et al*, 2006; Ulmert *et al*, 2009). When applied to select cancers, the accuracy of Osteopontin increases. Future research needs to assess whether the combination of Osteopontin with other markers can further improve its diagnostic value (Reinholz *et al*, 2002; O’Neill *et al*, 2005; Alonso *et al*, 2007; Ribeiro-Silva and Oliveira da Costa, 2008).

Meta-analysis has been a valuable tool in biomarker validation. One of its major limitations is the detection of true signals over the noise of heterogeneous input data. Categorical data analysis has a self-normalizing effect on study-to-study variations and may therefore be superior to conventional meta-regression algorithms. For the evaluation of Osteopontin as a biomarker for cancer, we have found conventional and categorical meta-analysis to be in agreement. This was not the case for the correlation of Osteopontin levels with tumor grade and stage (Figure 2 and Supplementary Figure S1). Here, the improved sensitivity of the categorical analysis is required to detect the existing trends in the published data sets.

## Figures and Tables

**Table 1:** Source references for data extraction

1	Bachmann IM, Ladstein RG, Straume O, Naumov GN, Akslen LA.	(2008)	*BMC Cancer*. 8, 362
2	Cappia S, Righi L, Mirabelli D, Ceppi P, Bacillo E, *et al.*	(2008)	*Am J Clin Pathol*. 130, 58
3	Carlos-Bregni R, Contreras E, Hiraki KR, Vargas PA, León JE, *et al.*	(2008)	*Oral Surg Oral Med Oral Pathol Oral Radiol Endod*. 105, e47
4	Carrer A, Zacchigna S, Balani A, Pistan V, Adami A, *et al.*	(2008)	*Eur J Cancer*. 44, 1761
5	Caruso DJ, Carmack AJ, Lokeshwar VB, Duncan RC, Soloway MS, *et al.*	(2008)	*Clin Cancer Res*. 14, 4111
6	Castellano G, Malaponte G, Mazzarino MC, Figini M, Marchese F, *et al.*	(2008)	*Clin Cancer Res*. 14, 7470
7	Chang PL, Harkins L, Hsieh YH, Hicks P, Sappayatosok K, *et al.*	(2008)	*J Histochem Cytochem*. 56, 57
8	Cho H, Hong SW, Oh YJ, Kim MA, Kang ES, *et al.*	(2008)	*J Cancer Res Clin Oncol*. 134, 909
9	Creaney J, Yeoman D, Demelker Y, Segal A, Musk AW, *et al.*	(2008)	*J Thorac Oncol*. 3, 851
10	Fredriksson S, Horecka J, Brustugun OT, Schlingemann J, Koong AC, *et al.*	(2008)	*Clin Chem*. 54, 582
11	Galamb O.	(2008)	*Orv Hetil*. 149, 1373. Hungarian.
12	Hui EP, Sung FL, Yu BK, Wong CS, Ma BB, *et al.*	(2008)	*Clin Cancer Res*. 14, 7080
13	Katase N, Tamamura R, Gunduz M, Murakami J, Asaumi J, *et al.*	(2008)	*Head Face Med*. 4, 28
14	Kato N, Motoyama T.	(2008)	*Histopathology*. 52, 682
15	Kittaka N, Takemasa I, Takeda Y, Marubashi S, Nagano H, *et al.*	(2008)	*Eur J Cancer*. 44, 885
16	Korita PV, Wakai T, Shirai Y, Matsuda Y, Sakata J, *et al.*	(2008)	*Hum Pathol*. 39, 1777
17	Lee CY, Tien HF, Hou HA, Chou WC, Lin LI.	(2008)	*Br J Haematol*.141, 736
18	Mack PC, Redman MW, Chansky K, Williamson SK, Farneth NC, *et al.*	(2008)	*J Clin Oncol*. 26, 4771
19	Matusan-Ilijas K, Behrem S, Jonjic N, Zarkovic K, Lucin K.	(2008)	*Pathol Oncol Res*. 14, 293
20	McAllister SS, Gifford AM, Greiner AL, Kelleher SP, Saelzler MP, *et al.*	(2008)	*Cell*. 133, 994
21	Mirza M, Shaughnessy E, Hurley JK, Vanpatten KA, Pestano GA, *et al.*	(2008)	*Int J Cancer*. 122, 889
22	Moore RG, Brown AK, Miller MC, Skates S, Allard WJ, *et al.*	(2008)	*Gynecol Oncol*. 108, 402
23	Mrochem J, Sodowski K, Deja R, Walaszek-Gruszka A, Wojcieszek A, *et al.*	(2008)	*Ginekol Pol*. 79, 271. Polish
24	Ohike N, Sato M, Kawahara M, Ohyama S, Morohoshi T.	(2008)	*JOP*. 9, 335
25	Oler G, Camacho CP, Hojaij FC, Michaluart P Jr, Riggins GJ, *et al.*	(2008)	*Clin Cancer Res*. 14, 4735
26	Patani N, Jiang W, Mokbel K.	(2008)	*Int J Cancer*. 122, 2646
27	Rangel J, Nosrati M, Torabian S, Shaikh L, Leong SP, *et al.*	(2008)	*Cancer*. 112, 144
28	Ribeiro-Silva A, Oliveira da Costa JP.	(2008)	*Int J Biol Markers*. 23, 154
29	Shimizu S, Tsukada J, Sugimoto T, Kikkawa N, Sasaki K, *et al.*	(2008)	*Int J Cancer*. 123, 1816
30	Tang H, Wang J, Bai F, Zhai H, Gao J, *et al.*	(2008)	*Cancer Invest*. 26, 60
31	Tun HW, Personett D, Baskerville KA, Menke DM, Jaeckle KA, *et al.*	(2008)	*Blood*. 111, 3200
32	Vergis R, Corbishley CM, Norman AR, Bartlett J, Jhavar S, *et al.*	(2008)	*Lancet Oncol*. 9, 342
33	Visintin I, Feng Z, Longton G, Ward DC, Alvero AB, *et al.*	(2008)	*Clin Cancer Res*. 14, 1065
34	Wang X, Chao L, Ma G, Chen L, Tian B, *et al.*	(2008)	*Eur J Clin Invest*. 38, 438
35	Wu IC, Wu MT, Chou SH, Yang SF, Goan YG, *et al.*	(2008)	*World J Surg*. 32, 1989
36	Yang GH, Fan J, Xu Y, Qiu SJ, Yang XR, *et al.*	(2008)	*Oncologist*. 13, 1155
37	Zdzisinska B, Bojarska-Junak A, Dmoszynska A, Kandefer-Szerszen M.	(2008)	*Arch Immunol Ther Exp (Warsz)*. 56, 207
38	Zhao J, Lu B, Xu H, Tong X, Wu G, *et al.*	(2008)	*Hepatology.* 48, 265
39	Zhao L, Li T, Wang Y, Pan Y, Ning H, *et al.*	(2008)	*Int J Clin Pract*. 62, 1056
40	Alonso SR, Tracey L, Ortiz P, Pérez-Gómez B, Palacios J, *et al.*	(2007)	*Cancer Res*. 67, 3450
41	Bao LH, Sakaguchi H, Fujimoto J, Tamaya T.	(2007)	*J Biomed Sci*. 14, 373
42	Bloomston M, Ellison EC, Muscarella P, Al-Saif O, Martin EW, *et al.*	(2007)	*Ann Surg Oncol*. 14, 211
43	Chandran UR, Ma C, Dhir R, Bisceglia M, Lyons-Weiler M, *et al.*	(2007)	*BMC Cancer*. 7, 64
44	Chang YS, Kim HJ, Chang J, Ahn CM, Kim SK, *et al.*	(2007)	*Lung Cancer*. 57, 373
45	Dai N, Bao Q, Lu A, Li J.	(2007)	*Oncology*. 72, 89
46	Del Sordo R, Cavaliere A, Sidoni A.	(2007)	*Am J Dermatopathol*. 29, 470
47	Dizdar O, Barista I, Kalyoncu U, Karadag O, Hascelik G, *et al.*	(2007)	*Am J Hematol*. 82, 185
48	Eto M, Kodama S, Nomi N, Uemura N, Suzuki M.	(2007)	*Auris Nasus Larynx*. 34, 343
49	Frey AB, Wali A, Pass H, Lonardo F.	(2007)	*Histopathology*. 50, 720
50	Gallot D, Marceau G, Laurichesse-Delmas H, Vanlieferinghen P, Dechelotte PJ, *et al.*	(2007)	*Fetal Diagn Ther*. 22, 161
51	Ghert M, Simunovic N, Cowan RW, Colterjohn N, Singh G.	(2007)	*Clin Orthop Relat Res*. 459, 8
52	Grigoriu BD, Scherpereel A, Devos P, Chahine B, Letourneux M, *et al.*	(2007)	*Clin Cancer Res*. 13, 2928
53	Grisaru D, Hauspy J, Prasad M, Albert M, Murphy KJ, *et al.*	(2007)	*Oncol Rep*. 18, 1347
54	Guglielmi G, Ciberti A, Foddis R, Ambrosino N, Chella A, *et al.*	(2007)	*G Ital Med Lav Ergon*. 29, 345. Italian
55	Gui SY, Li HH, Zuo L, Zhou Q, Wu Q, *et al.*	(2007)	*Zhonghua Yi Xue Za* *Zhi*. 87, 3219. Chinese
56	Higashiyama M, Ito T, Tanaka E, Shimada Y.	(2007)	*Ann Surg Oncol*. 14, 3419
57	Hu Z, Xiao T, Lin DM, Guo SP, Zhang ZQ, *et al.*	(2007)	*Zhonghua Zhong Liu Za Zhi*. 29, 591. Chinese
58	Jaeger J, Koczan D, Thiesen HJ, Ibrahim SM, Gross G, *et al.*	(2007)	*Clin Cancer Res*. 13, 806
59	Katakura A, Kamiyama I, Takano N, Shibahara T, Muramatsu T, *et al.*	(2007)	*Bull Tokyo Dent Coll*. 48, 199
60	Kuroda N, Hamaguchi N, Ohara M, Hirouchi T, Mizuno K, *et al.*	(2007)	*Med Mol Morphol*. 40, 218
61	Le QT, Kong C, Lavori PW, O’byrne K, Erler JT, *et al.*	(2007)	*Int J Radiat Oncol Biol Phys*. 69, 167
62	Lee YC, Pan HW, Peng SY, Lai PL, Kuo WS, *et al.*	(2007)	*Eur J Cancer*. 43, 736
63	Li Y, Lu Y, Ceng Y, Yang X.	(2007)	*Lin Chung Er Bi Yan Hou Tou Jing Wai Ke Za Zhi*. 21, 121. Chinese
64	Lin HM, Chatterjee A, Lin YH, Anjomshoaa A, Fukuzawa R, *et al.*	(2007)	*Oncol Rep.* 17, 1541
65	Matsuzaki H, Shima K, Muramatsu T, Ro Y, Hashimoto S, *et al.*	(2007)	*J Oral Pathol Med*. 36, 30
66	Meinhold-Heerlein I, Bauerschlag D, Zhou Y, Sapinoso LM, Ching K, *et al.*	(2007)	*Clin Cancer Res*. 13, 458
67	Mountzios G, Dimopoulos MA, Bamias A, Papadopoulos G, Kastritis E, *et al.*	(2007)	*Acta Oncol*. 46, 221
68	Mountzios G, Dimopoulos MA, Bamias A, Papadopoulos G, Kastritis E, *et al.*	(2007)	*Acta Oncol*. 46, 221
69	Nordsmark M, Eriksen JG, Gebski V, Alsner J, Horsman MR, *et al.*	(2007)	*Radiother Oncol*. 83, 389
70	Ogbureke KU, Nikitakis NG, Warburton G, Ord RA, Sauk JJ, *et al.*	(2007)	*Oral Oncol*. 43, 920
71	Pascaretti-Grizon F, Gaudin-Audrain C, Gallois Y, Retaillaud-Gaborit N, Baslé MF.	(2007)	*Morphologie*. 91, 180
72	Ramankulov A, Lein M, Kristiansen G, Loening SA, Jung K.	(2007)	*Prostate*. 67, 330
73	Ramankulov A, Lein M, Kristiansen G, Meyer HA, Loening SA, *et al.*	(2007)	*J Cancer Res Clin Oncol*. 133, 643
74	Reiniger IW, Wolf A, Welge-Lüssen U, Mueller AJ, Kampik A, *et al.*	(2007)	*Am J Ophthalmol*. 143, 705
75	Robbiani DF, Colon K, Ely S, Ely S, Chesi M, *et al.*	(2007)	*Hematol Oncol*. 25, 16
76	Rohde F, Rimkus C, Friederichs J, Rosenberg R, Marthen C, *et al.*	(2007)	*Int J Cancer*. 121, 1717
77	Said HM, Hagemann C, Staab A, Stojic J, Kühnel S, *et al.*	(2007)	*Radiother Oncol*. 83, 398
78	Sakaguchi H, Fujimoto J, Hong BL, Tamaya T.	(2007)	*Cancer Lett*. 247, 98
79	Shin HD, Park BL, Cheong HS, Yoon JH, Kim YJ, *et al.*	(2007)	*Int J Epidemiol*. 36, 1001
80	Soikkeli J, Lukk M, Nummela P, Virolainen S, Jahkola T, *et al.*	(2007)	*J Pathol*. 213, 180
81	Staibano S, Merolla F, Testa D, Iovine R, Mascolo M, *et al.*	(2007)	*Br J Cancer*. 97, 1545
82	Tigrani DY, Weydert JA.	(2007)	*Am J Clin Pathol*. 127, 580
83	Winfield HL, Kirkland F, Ramos-Ceballos FI, Horn TD.	(2007)	*Arch Dermatol*. 143, 1076
84	Wu CY, Wu MS, Chiang EP, Wu CC, Chen YJ, *et al.*	(2007)	*Gut*. 56, 782
85	Xie H, Song J, Du R, Liu K, Wang J, *et al.*	(2007)	*Dig Liver Dis*. 39, 167
86	Allan AL, George R, Vantyghem SA, Lee MW, Hodgson NC, *et al.*	(2006)	*Am J Pathol*. 169, 233
87	Bache M, Reddemann R, Said HM, Holzhausen HJ, Taubert H, *et al.*	(2006)	*Int J Radiat Oncol Biol Phys*. 66, 1481
88	Benoist-Lasselin C, de Margerie E, Gibbs L, Cormier S, Silve C, *et al.*	(2006)	*Bone*. 39, 17
89	Bramwell VH, Doig GS, Tuck AB, Wilson SM, Tonkin KS, *et al.*	(2006)	*Clin Cancer Res*. 12, 3337
90	Briese J, Schulte HM, Bamberger CM, Löning T, Bamberger AM.	(2006)	*Int J Gynecol Pathol*. 25, 161
91	Colin C, Baeza N, Bartoli C, Fina F, Eudes N, *et al.*	(2006)	*Oncogene*. 25, 2818
92	Dalla-Torre CA, Yoshimoto M, Lee CH, Joshua AM, de Toledo SR, *et al.*	(2006)	*BMC Cancer*. 6, 237
93	Darling MR, Gauthier M, Jackson-Boeters L, Daley TD, Chambers AF, *et al.*	(2006)	*Oral Oncol*. 42, 363
94	Debucquoy A, Goethals L, Geboes K, Roels S, Mc Bride WH, *et al.*	(2006)	*Radiother Oncol*. 80, 172
95	Feng HC, Tsao SW, Ngan HY, Kwan HS, Shih SM, *et al.*	(2006)	*Placenta*. 27, 521
96	Fluge Ø, Bruland O, Akslen LA, Lillehaug JR, Varhaug JE.	(2006)	*Thyroid*. 16, 161
97	Forootan SS, Foster CS, Aachi VR, Adamson J, Smith PH, *et al.*	(2006)	*Int J Cancer*. 118, 2255
98	Gong YH, Sun LP, Yuan Y.	(2006)	*Zhonghua Zhong Liu Za Zhi*. 28, 691. Chinese
99	Hashiguchi Y, Tsuda H, Bandera CA, Nishimura S, Inoue T, *et al.*	(2006)	*Med Oncol*. 23, 205
100	Huang J, Sheng HH, Shen T, Hu YJ, Xiao HS, *et al.*	(2006)	*FEBS Lett*. 580, 3571
101	Kadkol SS, Lin AY, Barak V, Kalickman I, Leach L, *et al.*	(2006)	*Invest Ophthalmol Vis Sci*. 47, 802
102	Kim J, Ki SS, Lee SD, Han CJ, Kim YC, *et al.*	(2006)	*Am J Gastroenterol*. 101, 2051
103	Kita Y, Natsugoe S, Okumura H, Matsumoto M, Uchikado Y, *et al.*	(2006)	*Br J Cancer*. 95, 634
104	Köbel M, Langhammer T, Hüttelmaier S, Schmitt WD, Kriese K, *et al.*	(2006)	*Mod Pathol*. 19, 581
105	Koopmann J, Rosenzweig CN, Zhang Z, Canto MI, Brown DA, *et al.*	(2006)	*Clin Cancer Res*. 12, 442
106	Le QT, Chen E, Salim A, Cao H, Kong CS, *et al.*	(2006)	*Clin Cancer Res*. 12, 1507
107	Luo JH, Ren B, Keryanov S, Tseng GC, Rao UN, *et al.*	(2006)	*Hepatology*. 44, 1012
108	Matusan K, Dordevic G, Stipic D, Mozetic V, Lucin K.	(2006)	*J Surg Oncol*. 94, 325
109	Miller CT, Lin L, Casper AM, Lim J, Thomas DG, *et al.*	(2006)	*Oncogene*. 25, 409
110	Nakae M, Iwamoto I, Fujino T, Maehata Y, Togami S, *et al.*	(2006)	*J Obstet Gynaecol Res*. 32, 309
111	Petrik D, Lavori PW, Cao H, Zhu Y, Wong P, *et al.*	(2006)	*J Clin Oncol.* 24, 5291
112	Rogers CD, Fukushima N, Sato N, Shi C, Prasad N, *et al.*	(2006)	*Cancer Biol Ther*. 5, 1383
113	Rudland S, Martin L, Roshanlall C, Winstanley J, Leinster S, *et al.*	(2006)	*Clin Cancer Res*. 12, 1192
114	Sando N, Oka K, Moriya T, Saito H, Nagakura S, *et al.*	(2006)	*APMIS*. 114, 581
115	Vordermark D, Said HM, Katzer A, Kuhnt T, Hänsgen G, *et al.*	(2006)	*BMC Cancer*. 6, 207
116	Wang G, Platt-Higgins A, Carroll J, de Silva Rudland S, Winstanley J, *et al.*	(2006)	*Cancer Res*. 66, 1199
117	Wong YF, Cheung TH, Tsao GS, Lo KW, Yim SF, *et al.*	(2006)	*Int J Cancer*. 118, 2461
118	Ye B, Skates S, Mok SC, Horick NK, Rosenberg HF, *et al.*	(2006)	*Clin Cancer Res*. 12, 432
119	Yuan RH, Jeng YM, Chen HL, Lai PL, Pan HW, *et al.*	(2006)	*J Pathol*. 209, 549
120	Zhang H, Ye QH, Ren N, Zhao L, Wang YF, *et al.*	(2006)	*J Cancer Res Clin Oncol*. 132, 709
121	Zhang HZ, Liu JG, Wei YP, Wu C, Cao YK, *et al.*	(2006)	*Nan Fang Yi Ke Da Xue Xue Bao*. 26, 1612. Chinese
122	Ang C, Chambers AF, Tuck AB, Winquist E, Izawa JI.	(2005)	*BJU Int*. 96, 803
123	Boldrini L, Donati V, Dell’Omodarme M, Prati MC, Faviana P, *et al.*	(2005)	*Br J Cancer*. 93, 453
124	Bramwell VH, Tuck AB, Wilson SM, Stitt LW, Cherian AK, *et al.*	(2005)	*Cancer Biol Ther*. 4, 1336
125	Briese J, Oberndörfer M, Schulte HM, Löning T, Bamberger AM.	(2005)	*Int J Gynecol Pathol*. 24, 271
126	Celetti A, Testa D, Staibano S, Merolla F, Guarino V, *et al.*	(2005)	*Clin Cancer Res*. 11, 8019
127	Donati V, Boldrini L, Dell’Omodarme M, Prati MC, Faviana P, *et al.*	(2005)	*Clin Cancer Res*. 11, 6459
128	Guarino V, Faviana P, Salvatore G, Castellone MD, Cirafici AM, *et al.*	(2005)	*J Clin Endocrinol Metab*. 90, 5270
129	Hoshi N, Sugino T, Suzuki T.	(2005)	*Pathol Int*. 55, 484
130	Hu Z, Lin D, Yuan J, Xiao T, Zhang H, *et al.*	(2005)	*Clin Cancer Res*. 11, 4646
131	Iso Y, Sawada T, Okada T, Kubota K.	(2005)	*J Surg Oncol*. 92, 304
132	Kao CL, Chiou SH, Chen YJ, Singh S, Lin HT, *et al.*	(2005)	*Mod Pathol*. 18, 769
133	Kao CL, Chiou SH, Ho DM, Chen YJ, Liu RS, *et al.*	(2005)	*Am J Clin Pathol*. 123, 297
134	Kawakami T, Shimizu T, Kimura A, Hasegawa H, Siar CH, *et al.*	(2005)	*Eur J Med Res*. 10, 475
135	Libra M, Indelicato M, De Re V, Zignego AL, Chiocchetti A, *et al.*	(2005)	*Cancer Biol Ther*. 4, 1192
136	Matusan K, Dordevic G, Mozetic V, Lucin K.	(2005)	*Pathol Oncol Res*. 11, 108
137	Mor G, Visintin I, Lai Y, Zhao H, Schwartz P, *et al.*	(2005)	*Proc Natl Acad Sci USA.* 102, 7677
138	O’Neill CJ, Deavers MT, Malpica A, Foster H, McCluggage WG.	(2005)	*Am J Surg Pathol*. 29, 1034
139	Overgaard J, Eriksen JG, Nordsmark M, Alsner J, Horsman MR, *et al.*	(2005)	*Lancet Oncol*. 6, 757
140	Pass HI, Lott D, Lonardo F, Harbut M, Liu Z, *et al.*	(2005)	*N Engl J Med*. 353, 1564
141	Peng SY, Ou YH, Chen WJ, Li HY, Liu SH, *et al.*	(2005)	*Int J Oncol*. 26, 1053
142	Rosen DG, Wang L, Atkinson JN, Yu Y, Lu KH, *et al.*	(2005)	*Gynecol Oncol*. 99, 267
143	Sedivy R, Kalipciyan M, Mazal PR, Wolf B, Wrba F, *et al.*	(2005)	*Cancer Detect Prev*. 29, 8
144	Sedivy R, Peters K, Klöppel G.	(2005)	*Virchows Arch*. 446, 41
145	Shimada Y, Watanabe G, Kawamura J, Soma T, Okabe M, *et al.*	(2005)	*Oncology*. 68, 285
146	Sun XJ, Zuo WS, Ma H, Hou WH, Cai SP, *et al.*	(2005)	*Zhonghua Zhong Liu Za Zhi*. 27, 292. Chinese
147	Terpos E, Mihou D, Szydlo R, Tsimirika K, Karkantaris C, *et al.*	(2005)	*Leukemia*. 19, 1969
148	Wong TS, Kwong DL, Sham J, Wei WI, Kwong YL, *et al.*	(2005)	*Eur J Surg Oncol*. 31, 555
149	Zhang DT, Yuan J, Yang L, Guo XN, Hao ZM, *et al.*	(2005)	*Zhonghua Zhong Liu Za Zhi*. 27, 167. Chinese
150	Zhang H, Ren N, Ye QH, Sun HC, Wang L, *et al.*	(2005)	*Zhonghua Wai Ke Za Zhi*. 43, 985. Chinese
151	Zhou Y, Dai DL, Martinka M, Su M, Zhang Y, *et al.*	(2005)	*J Invest Dermatol*. 124, 1044
152	Brakora KA, Lee H, Yusuf R, Sullivan L, Harris A, *et al.*	(2004)	*Gynecol Oncol*. 93, 361
153	Coppola D, Szabo M, Boulware D, Muraca P, Alsarraj M, *et al.*	(2004)	*Clin Cancer Res*. 10, 184
154	Fisher LW, Jain A, Tayback M, Fedarko NS.	(2004)	*Clin Cancer Res*. 10, 8501
155	Koopmann J, Fedarko NS, Jain A, Maitra A, Iacobuzio-Donahue C, *et al.*	(2004)	*Cancer Epidemiol Biomarkers Prev*. 13, 487
156	Lu KH, Patterson AP, Wang L, Marquez RT, Atkinson EN, *et al.*	(2004)	*Clin Cancer Res*. 10, 3291
157	Maki M, Athanasou N.	(2004)	*Hum Pathol*. 35, 69
158	Martinetti A, Bajetta E, Ferrari L, Zilembo N, Seregni E, *et al.*	(2004)	*Endocr Relat Cancer*. 11, 771
159	Santin AD, Zhan F, Bellone S, Palmieri M, Cane S, *et al.*	(2004)	*Int J Cancer*. 112, 14
160	Schneider S, Yochim J, Brabender J, Uchida K, Danenberg KD, *et al.*	(2004)	*Clin Cancer Res*. 10, 1588
161	Schorge JO, Drake RD, Lee H, Skates SJ, Rajanbabu R, *et al.*	(2004)	*Clin Cancer Res*. 10, 3474
162	Standal T, Hjorth-Hansen H, Rasmussen T, Dahl IM, Lenhoff S, *et al.*	(2004)	*Haematologica*. 89, 174
163	Terashi T, Aishima S, Taguchi K, Asayama Y, Sugimachi K, *et al.*	(2004)	*Liver Int*. 24, 38
164	Van Heek NT, Maitra A, Koopmann J, Fedarko N, Jain A, *et al.*	(2004)	*Cancer Biol Ther*. 3, 651
165	Yamagishi SI, Suzuki T, Ohkuro H, Yagihashi S.	(2004)	*J Endocrinol Invest*. 27, 870
166	Agrawal D, Chen T, Irby R, Quackenbush J, Chambers AF, *et al.*	(2003)	*C R Biol*. 326, 1041
167	Batorfi J, Fulop V, Kim JH, Genest DR, Doszpod J, *et al.*	(2003)	*Gynecol Oncol*. 89, 134
168	Carlinfante G, Vassiliou D, Svensson O, Wendel M, Heinegård D, *et al.*	(2003)	*Clin Exp Metastasis*. 20, 437
169	Giordano TJ, Thomas DG, Kuick R, Lizyness M, Misek DE, *et al.*	(2003)	*Am J Pathol*. 162, 521
170	Korkola JE, DeVries S, Fridlyand J, Hwang ES, Estep AL, *et al.*	(2003)	*Cancer Res*. 63, 7167
171	Le QT, Sutphin PD, Raychaudhuri S, Yu SC, Terris DJ, *et al.*	(2003)	*Clin Cancer Res*. 9, 59
172	Pan HW, Ou YH, Peng SY, Liu SH, Lai PL, *et al.*	(2003)	*Cancer*. 98, 119
173	Saeki Y, Mima T, Ishii T, Ogata A, Kobayashi H, *et al.*	(2003)	*Br J Haematol*. 123, 263
174	Varnum SM, Covington CC, Woodbury RL, Petritis K, Kangas LJ, *et al.*	(2003)	*Breast Cancer Res Treat*. 80, 87
175	Wang-Rodriguez J, Urquidi V, Rivard A, Goodison S.	(2003)	*Breast Cancer Res*. 5, R136
176	Ye QH, Qin LX, Forgues M, He P, Kim JW, *et al.*	(2003)	*Nat Med*. 9, 416
177	Agrawal D, Chen T, Irby R, Quackenbush J, Chambers AF, *et al.*	(2002)	*J Natl Cancer Inst*. 94, 513
178	Ding L, Zheng S, Cao J.	(2002)	*Zhonghua Yi Xue Za Zhi*. 82, 970. Chinese
179	Ding L, Zheng S.	(2002)	*Zhonghua Wai Ke Za Zhi*. 40, 773. Chinese
180	Gotoh M, Sakamoto M, Kanetaka K, Chuuma M, Hirohashi S.	(2002)	*Pathol Int*. 52, 19
181	Hotte SJ, Winquist EW, Stitt L, Wilson SM, Chambers AF.	(2002)	*Cancer*. 95, 506
182	Kim JH, Skates SJ, Uede T, Wong KK, Schorge JO, *et al.*	(2002)	*JAMA*. 287, 1671
183	Lee S, Baek M, Yang H, Bang YJ, Kim WH, *et al.*	(2002)	*Cancer Lett*. 184, 197
184	Oyama T, Sano T, Hikino T, Xue Q, Iijima K, *et al.*	(2002)	*Virchows Arch*. 440, 267
185	Reinholz MM, Iturria SJ, Ingle JN, Roche PC.	(2002)	*Breast Cancer Res Treat*. 74, 255
186	Rudland PS, Platt-Higgins A, El-Tanani M, De Silva Rudland S, *et al.*	(2002)	*Cancer Res*. 62, 3417
187	Sulzbacher I, Birner P, Trieb K, Lang S, Chott A.	(2002)	*Virchows Arch*. 441, 345
188	Tókés AM, Krausz J, Kulka J, Jäckel M, Kádár A.	(2002)	*Orv Hetil*. 143, 1841 Hungarian
189	Cummings TJ, Hulette CM, Bigner SH, Riggins GJ, McLendon RE.	(2001)	*Arch Pathol Lab Med*. 125, 637
190	Fedarko NS, Jain A, Karadag A, Van Eman MR, Fisher LW.	(2001)	*Clin Cancer Res*. 7, 4060
191	Gaumann A, Petrow P, Mentzel T, Mayer E, Dahm M, *et al.*	(2001)	*Virchows Arch*. 439, 668
192	Kawahara K, Niguma T, Yoshino T, Omonishi K, Hatakeyama S, *et al.*	(2001)	*Pathol Int*. 51, 718
193	Markert JM, Fuller CM, Gillespie GY, Bubien JK, McLean LA, *et al.*	(2001)	*Physiol Genomics*. 5, 21
194	Matsuzaka K, Inoue T, Nashimoto M, Takemoto K, Ishikawa H, *et al.*	(2001)	*Bull Tokyo Dent Coll*. 42, 51
195	Mok SC, Chao J, Skates S, Wong K, Yiu GK, *et al.*	(2001)	*J Natl Cancer Inst*. 93, 1458
196	Nishie A, Masuda K, Otsubo M, Migita T, Tsuneyoshi M, *et al.*	(2001)	*Clin Cancer Res*. 7, 2145
197	Zhang J, Takahashi K, Takahashi F, Shimizu K, Ohshita F, *et al.*	(2001)	*Cancer Lett*. 171, 215
198	Fisher JL, Field CL, Zhou H, Harris TL, Henderson MA, *et al.*	(2000)	*Breast Cancer Res Treat*. 61, 1
199	Ibrahim T, Leong I, Sanchez-Sweatman O, Khokha R, Sodek J, *et al.*	(2000)	*Clin Exp Metastasis*. 18, 253
200	Jin Y, Kuroda N, Kakiuchi S, Yamasaki Y, Miyazaki E, *et al.*	(2000)	*Pathol Int*. 50, 421
201	Maki M, Hirota S, Kaneko Y, Morohoshi T.	(2000)	*Pathol Int*. 50, 531
202	Oyama T, Iijima K, Takei H, Horiguchi J, Iino Y, *et al.*	(2000)	*Breast Cancer*. 7, 326
203	Takano S, Tsuboi K, Tomono Y, Mitsui Y, Nose T.	(2000)	*Br J Cancer*. 82, 1967
204	Wong IH, Chan AT, Johnson PJ.	(2000)	*Clin Cancer Res*. 6, 2183
205	Devoll RE, Li W, Woods KV, Pinero GJ, Butler WT, *et al.*	(1999)	*J Oral Pathol Med*. 28, 97
206	Kusafuka K, Yamaguchi A, Kayano T, Takemura T.	(1999)	*Pathol Res Pract*. 195, 733
207	Sharp JA, Sung V, Slavin J, Thompson EW, Henderson MA.	(1999)	*Lab Invest*. 79, 869
208	Shijubo N, Uede T, Kon S, Maeda M, Segawa T, *et al.*	(1999)	*Am J Respir Crit Care Med*. 160, 1269
209	Thalmann GN, Sikes RA, Devoll RE, Kiefer JA, Markwalder R, *et al.*	(1999)	*Clin Cancer Res*. 5, 2271
210	Tozawa K, Yamada Y, Kawai N, Okamura T, Ueda K, *et al.*	(1999)	*Urol Int*. 62, 155
211	Kim YW, Park YK, Lee J, Ko SW, Yang MH.	(1998)	*J Korean Med Sci*. 13, 652
212	Tiniakos DG, Yu H, Liapis H.	(1998)	*Hum Pathol*. 29, 1250
213	Tuck AB, O’Malley FP, Singhal H, Harris JF, Tonkin KS, *et al.*	(1998)	*Int J Cancer*. 79, 502
214	Tunio GM, Hirota S, Nomura S, Kitamura Y.	(1998)	*Arch Pathol Lab Med.* 1122, 1087
215	Ue T, Yokozaki H, Kitadai Y, Yamamoto S, Yasui W, *et al.*	(1998)	*Int J Cancer*. 79, 127
216	Casson AG, Wilson SM, McCart JA, O’Malley FP, Ozcelik H, *et al.*	(1997)	*Int J Cancer*. 72, 739
217	Gillespie MT, Thomas RJ, Pu ZY, Zhou H, Martin TJ, *et al.*	(1997)	*Int J Cancer*. 73, 812
218	Gladson CL, Dennis C, Rotolo TC, Kelly DR, Grammer JR.	(1997)	*Am J Pathol*. 150, 1631
219	Singhal H, Bautista DS, Tonkin KS, O’Malley FP, Tuck AB, *et al.*	(1997)	*Clin Cancer Res*. 3, 605
220	Tuck AB, O’Malley FP, Singhal H, Tonkin KS, Harris JF, *et al.*	(1997)	*Arch Pathol Lab Med.* 121, 578
221	Bautista DS, Denstedt J, Chambers AF, Harris JF.	(1996)	*J Cell Biochem*. 61, 402
222	Chambers AF, Wilson SM, Kerkvliet N, O’Malley FP, Harris JF, *et al.*	(1996)	*Lung Cancer*. 15, 311
223	Bellahcène A, Castronovo V.	(1995)	*Am J Pathol.* 146, 95
224	Hirota S, Asada H, Kohri K, Tsukamoto Y, Ito A, *et al.*	(1995)	*J Invest Dermatol.* 105, 138
225	Hirota S, Ito A, Nagoshi J, Takeda M, Kurata A, *et al.*	(1995)	*Lab Invest*. 72, 64
226	Saitoh Y, Kuratsu J, Takeshima H, Yamamoto S, Ushio Y.	(1995)	*Lab Invest*. 72, 55
227	Brown LF, Papadopoulos-Sergiou A, Berse B, Manseau EJ, Tognazzi K, *et al.*	(1994)	*Am J Pathol*. 145, 610
228	Senger DR, Perruzzi CA, Papadopoulos A.	(1989)	*Anticancer Res*. 9, 1291

PubMed references for ‘osteopontin AND cancer’ were screened for studies involving human subjects and then filtered for the presentation of original data. This left 228 publications to be used for data extraction. The references are listed in reverse chronologic order. For space considerations, titles are omitted. Foreign language articles are indicated as such.

**Table 2:** Osteopontin and survival in individual cancers

**Cancer**	**Concordance**	***P*-value**	**Studies**
Lung	1.000	**0.001**	3
Breast	0.917	**0.004**	8
Prostate	1.000	**0.013**	3
Head and neck	1.000	**0.020**	4
Liver	0.875	**0.033**	8
Cervical	1.000	0.126	3
Esophageal	1.000	0.126	3
Gastric	0.750	0.189	3
Kidney	1.000	0.249	2
Mesothelioma	1.000	0.249	2

Published curves for overall survival were digitised for analysis. *P*-values in bold are considered significant. They indicate that Osteopontin over-expression is associated with elevated risk for death from cancer. For several cancer types, only one published study was available. Those cases were excluded from the meta-analysis. Shown are only cancers for which more than one published study was available for evaluation.

**Table 3:** Osteopontin and survival in distinct clinical specimens

	**Number of studies**	**Concordance**	***P*-value**
*Specimen*			
*(A) All tumors combined*			
Tumor	31	0.825	**<0.0001**
Plasma	14	1	**<0.0001**
Serum	3	1	**0.04**
			
*Cancer type*			
*(B) Plasma, individual cancers*			
Lung	1	1	**0.00**
Prostate	2	1	**0.08**
Breast	2	1	**0.08**
Head and neck	3	1	0.13
			
*Cancer type*			
*(C) Tumor, individual cancers*			
Liver	7	0.857	**0.06**
Breast	5	0.800	0.19
Esophageal	2	1	0.24
Head and neck	2	1	0.25
Lung	2	1	0.25
Cervical	2	1	0.25
Gastric	2	0.714	0.28

The concordance and probability of error were calculated for the null-hypothesis that Osteopontin levels are not correlated with high risk for short survival. (A) All tumors combined in distinct types of clinical samples. (B) Plasma Osteopontin in individual cancers (for serum Osteopontin see main text). (C) Tumor Osteopontin in individual cancers. Bold values indicate *P*<0.1

**Table 4:** Categorical meta-analysis of tumor grade and stage

**Cancer type**	**Studies**	**Patients**	**Pearson *P*-value**	**Linear *P*-value**	**Correlation coefficient**	**Non-linear *P*-value**
*(A) Tumor grade*						
All	42	4408	<0.001	<0.001	0.27	<0.001
Breast	6	1061	<0.001	<0.001	0.28	<0.001
Endometrial	3	236	<0.001	0.004	−0.19	<0.001
Esophageal	2	161	<0.001	0.001	−0.26	<0.001
Gastric	3	428	<0.001	<0.001	−0.65	<0.001
Glioma	5	180	<0.001	<0.001	0.89	<0.001
Head & neck	2	92	<0.001	<0.001	0.59	<0.001
Liver	6	870	<0.001	<0.001	0.72	<0.001
Lung	4	610	<0.001	<0.001	−0.24	<0.001
Oral	3	103	<0.001	0.170	0.14	<0.001
Ovarian	5	379	<0.001	<0.001	0.68	<0.001
Prostate	2	117	<0.001	<0.001	0.45	<0.001
Renal	1	171	<0.001	<0.001	1	1
						
*(B) Tumor stage (T)*						
All	56	4480	<0.001	<0.001	0.70	<0.001
Breast	3	236	<0.001	0.003	0.20	<0.001
Cervical	2	170	0.416	0.417	−0.06	N/A
Colorectal	6	420	<0.001	<0.001	0.84	<0.001
Endometrial	4	122	<0.001	0.052	−0.18	<0.001
Esophageal	6	284	<0.001	<0.001	0.67	<0.001
Gastric	8	772	<0.001	<0.001	0.85	<0.001
Head & neck	5	569	<0.001	<0.001	1.00	N/A
Liver	4	497	<0.001	<0.001	0.89	<0.001
Lung	5	692	<0.001	<0.001	0.96	<0.001
Myeloma	1	30	<0.001	<0.001	1.00	N/A
Oral	1	26	<0.001	<0.001	−1.00	1
Ovarian	8	444	<0.001	<0.001	0.22	<0.001
Prostate	1	47	<0.001	<0.001	−1.00	1
Renal	1	171	<0.001	<0.001	1.00	N/A
						
*(C) Tumor stage (N)*						
All	27	3159	<0.001	<0.001	0.81	<0.001
Breast	7	909	<0.001	<0.001	0.59	N/A
Esophageal	3	336	<0.001	<0.001	1.00	N/A
Gastric	7	1013	<0.001	<0.001	1.00	N/A
Head & neck	4	469	<0.001	<0.001	1.00	N/A
Liver	2	145	0.055	0.056	−0.16	N/A
Lung	1	130	<0.001	<0.001	0.43	<0.001
Melanoma	1	68	<0.001	<0.001	1.00	N/A
Oral	1	46	<0.001	<0.001	1.00	N/A
Renal	1	43	<0.001	<0.001	1.00	N/A
						
*(D) Tumor stage (M)*						
All	28	1900	<0.001	<0.001	0.72	N/A
Bladder	1	23	<0.001	<0.001	1.00	N/A
Breast	3	102	<0.001	<0.001	0.34	N/A
Colorectal	1	10	0.002	0.003	1.00	N/A
Gastric	4	612	<0.001	<0.001	1.00	N/A
Head & neck	3	113	<0.001	<0.001	1.00	N/A
Liver	5	187	<0.001	<0.001	−0.40	N/A
Lung	6	644	<0.001	<0.001	0.75	N/A
Melanoma	2	43	<0.001	<0.001	1.00	N/A
Oral	1	26	<0.001	<0.001	1.00	N/A
Prostate	1	10	0.002	0.003	1.00	N/A
Thyroid	1	130	<0.001	<0.001	1.00	N/A

Published Osteopontin levels in relation to tumor grade or tumor stage were analyzed.

As a test for independence of the ranked data we used the Pearson *χ*^2^-test. To assess linear and non-linear trends of the ranked data we applied the Mantel–Haenszel *χ*^2^-test. N/A indicates that there were only two outcomes, and a non-linear fit is not measurable.

**Table 5:** Categorical meta-analysis of tumor progression

**Cancer Type**	**Studies**	**Patients**	**Pearson *P*-value**	**Linear *P*-value**	**Correlation coefficient**	**Non-linear *P*-value**
All	34	2425	<0.001	<0.001	0.68	<0.001
Breast	4	172	<0.001	<0.001	0.75	<0.001
Cervical	1	398	<0.001	<0.001	1.00	1
Esophageal	1	46	<0.001	<0.001	0.77	<0.001
Gestational Trophoblastic tumor	4	86	<0.001	<0.001	−1.00	N/A
Head and neck	1	82	<0.001	<0.001	1.00	1
Liver	7	731	<0.001	<0.001	0.68	N/A
Mesothelioma	3	148	<0.001	<0.001	1.00	1
Myeloma	3	208	<0.001	<0.001	1.00	1
Non-mel.	1	36	<0.001	<0.001	−1.00	N/A
Oral	2	230	<0.001	<0.001	0.86	<0.001
Ovarian	5	213	<0.001	<0.001	−0.80	<0.001
Prostate	2	75	<0.001	<0.001	1	N/A

In the early stages of transformation, tumor progression can be described as the transition from normal tissue to precancerous lesions (dysplasia, metaplasia), preinvasive cancer, and cancer. The ranked levels of Osteopontin expression are significantly associated with the progression of liver cancer, myeloma, head and neck cancer, cervical cancer, prostate cancer, oral cancer, breast cancer, and mesothelioma. Unexpectedly, the meta-analysis reveals an inverse correlation to the progression of skin cancer and gestational trophoblastic tumor. Non-mel.=non-melanoma skin cancer.
