# Supplementary material for: Osteopontin is a marker for cancer aggressiveness and patient survival
Source: Br J Cancer. 2010 Sep 7;103(6):861–9. doi: 10.1038/sj.bjc.6605834 (PMC2966627; doi:10.1038/sj.bjc.6605834)
Supplement: Supplementary Information [file 6605834x1.doc]

**Supplement 1:**

Conventional meta-analysis of survival We extracted microarray data for the Spp1 gene (Osteopontin) from Oncomine (Rhodes *et al*, 2004) with no threshold for gene rank, a threshold of 0.001 for p-value, and limited to mRNA arrays (cutoff 10/2009). The meta-analysis function contained in the software (Oncomine 4.2, www.oncomine.com) was applied. Various data sets were compared according to the rank for a gene, which is the median rank for that gene across each of the analyses. The p-value for a gene is its p-value for the median-ranked analysis. Meta-analysis from the literature may be compromised by publication bias in favor of significant differences between study group and control group (the “file drawer problem”). As the microarray data were deposited without specific focus on Osteopontin, the evaluation of the Oncomine data can control for potential bias in the evaluation of the literature data.

In Oncomine, elevated Osteopontin levels were associated with death in 1-5 years in brain cancer (Table S1), head and neck cancer (p = 0.026, n = 34), and colorectal cancer (p = 0.042, n = 94), but not with other cancers investigated. The results for head and neck cancer and colorectal cancer rely on only one study each. In contrast to Oncomine, our categorical meta-analysis using published results identified Osteopontin as also significantly associated with short survival in breast, lung, and prostate cancers. Neither the literature data nor the Oncomine data indicated a prognostic value for Osteopontin in renal cancer.

**Table S1: Osteopontin and survival in individual cancers.** Separate probabilities are calculated for Osteopontin over-expression and Osteopontin under-expression as a predictor of survival. P-values in bold are considered significant. They indicate that Osteopontin over-expression is associated with death in 1-5 years in brain cancer. Shown are only cancers for which more than one study was available for evaluation.

|  |  | **p-value** | **p-value** |  |  |
| --- | --- | --- | --- | --- | --- |
| **cancer** |  | **over** | **under** | **n** | **data sets** |
|  |  |  |  |  |  |
| **brain** |  | **0.039** | 0.847 | 479 | 8 |
| **sarcoma** |  | 0.084 | 0.907 | 34 | 2 |
| **prostate** |  | 0.087 | 0.941 | 685 | 2 |
| **leukemia** |  | 0.109 | 0.968 | 309 | 4 |
| **myeloma** |  | 0.163 | 0.293 | 743 | 3 |
| **lung** |  | 0.186 | 0.814 | 988 | 6 |
| **bladder** |  | 0.287 | 0.539 | 99 | 2 |
| **breast** |  | 0.303 | 0.497 | 1125 | 6 |
| **kidney** |  | 0.452 | 0.401 | 391 | 3 |
| **lymphoma** |  | 0.671 | 0.124 | 1279 | 7 |
| **ovaries** |  | 0.682 | 0.532 | 445 | 3 |
| **melanoma** |  | 0.794 | 0.089 | 185 | 2 |

**Supplement 2:**

One traditional technique of meta-analysis is the determination of effect sizes between two variables. We used Cohen’s d (Thalheimer/Cook; Cohen 1992) to measure effect size, calculated according to Equation 1, where the subscripts refer to two distinct sets of patients differing by grade or stage, x is the mean value for the set, n is the number of patients in the set, and s is the standard deviation. When calculating the mean and standard deviation of the Osteopontin values for each set, the sample size for each study contributing to that set was used as a weight.

Equation 1

We analyzed published Osteopontin immunohistochemistry scores in relation to tumor grade or stage by conventional meta-analysis using weighted averages. The calculated effect sizes (Cohen’s d) for each pair of outcomes did not reveal a clear trend (Figure S1).

**Figure S1:** **Correlation of tumor grade and stage with Osteopontin immunohistochemistry scores.** We evaluated Osteopontin as a marker for stage and grade with a conventional meta-analysis approach. **A)** Each yellow circle represents one group of patients reported in a single publication. The solid blue dots show the weighted mean Osteopontin immunohistochemistry scores, with the number of patients in each group used as the weight. The blue lines indicate the 95% confidence intervals for the immunohistochemistry scores at each grade or stage. **B)** An alternative to using Pearson’s r for assessing effect size is Cohen’s d (Thalheimer/Cook), which is constructed by examining the difference in two population means, normalized by their pooled standard deviation. The measure assumes that effect sizes of 0.20 are small, 0.50 are medium, and 0.80 or greater are large. In the case of a positive correlation between outcome and Osteopontin score, one would anticipate a trend of increasing effect size with increasing difference in outcome. Deviations from this expectation occur when grade 4 or stage 4 samples are involved, which may be due to insufficient power. The smallest number of groups was reported for level 4 in both stage and grade. **C)** We applied a two-tailed, heteroscedastic Student’s t test to the data in Figure S1A and found that the difference in the means was only significant for the comparison of grades 1 and 3. The numbers represent significance values. The challenge in applying these techniques more generally is combining the disparate types of results that comprise the wider data set. Ranking addresses this problem by letting the studies be self-normalizing.

**
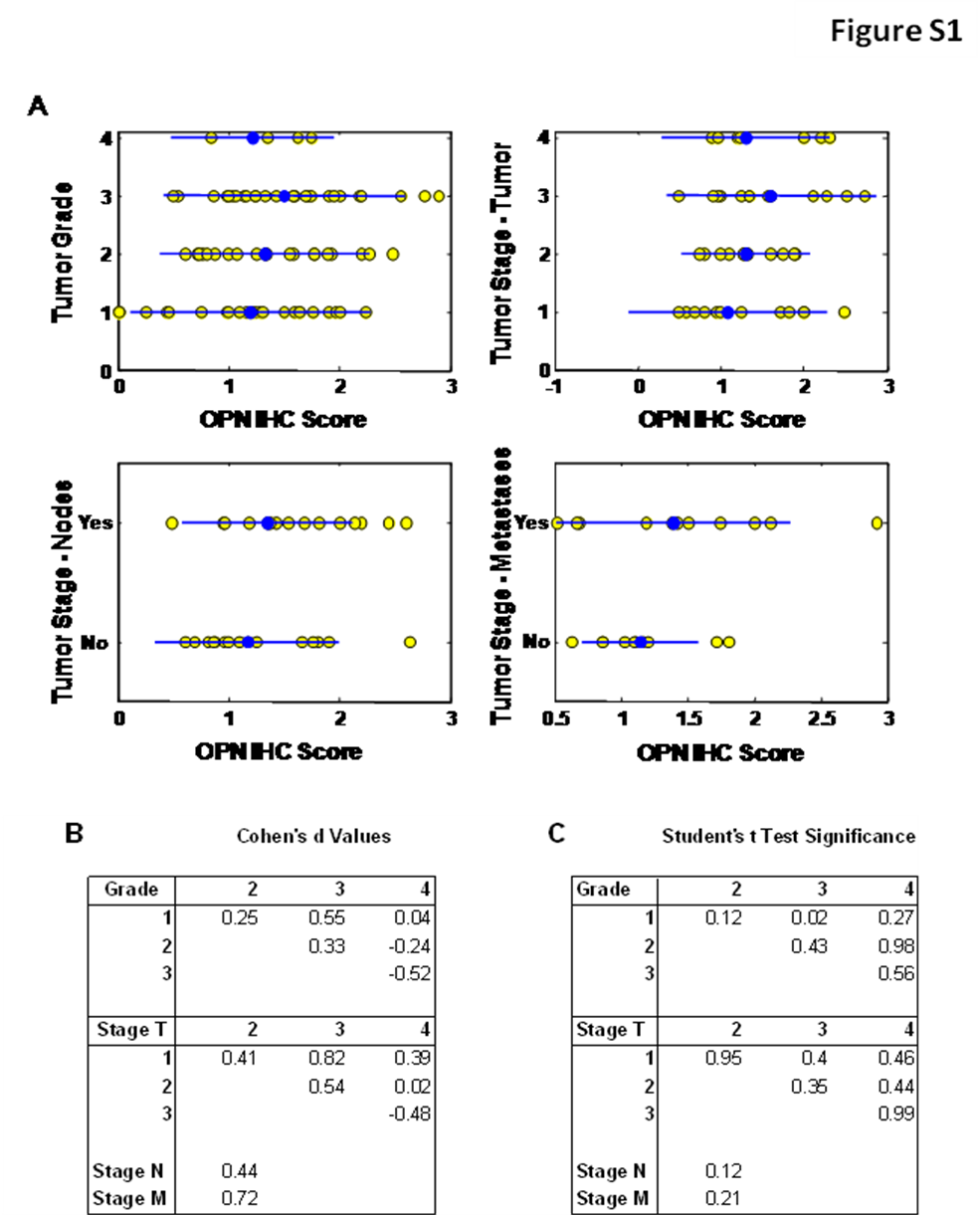
**

**Supplement References**

Cohen J (1992) A power primer. *Psychol Bull* **112** 155-159.

Rhodes DR, Yu J, Shanker K, Deshpande N, Varambally R, Ghosh D, Barrette T, Pandey A, Chinnaiyan AM (2004) ONCOMINE: a cancer microarray database and integrated data-mining platform. *Neoplasia* **6** 1-6.

Thalheimer W, Cook S. How to calculate effect sizes from published research articles: A simplified methodology. http://www.work-learning.com/effect_sizes.htm
